# Nursing students' perceptions of teachers' uncivil behaviors: a qualitative research

**Published:** 2017-11-05

**Authors:** Anahita Masoumpoor, Fariba Borhani, Abbas Abbaszadeh, Maryam Rassouli

**Affiliations:** 1 *Department of Pediatric Nursing, School of Nursing and Midwifery, Shahid Beheshti University of Medical Sciences, Tehran, Iran. *; 2 *Associate Professor, Medical Ethics and Law Research Center, Department of Medical-Surgical Nursing, School of Nursing and Midwifery, Shahid Beheshti University of Medical Sciences, Tehran, Iran. *; 3 *Professor, Department of Medical-Surgical Nursing, School of Nursing and Midwifery, Shahid Beheshti University of Medical Sciences, Tehran, Iran. *; 4 *Associate Professor, Department of Pediatric Nursing, School of Nursing and Midwifery, Shahid Beheshti University of Medical Sciences, Tehran, Iran.*

**Keywords:** *Uncivil behavior*, *Student perception*, *Nursing teachers*, *Qualitative research*

## Abstract

One of the main issues in nursing education that teachers and students frequently encounter is uncivil behaviors. This type of behavior is destructive for the teaching and learning environment. As teachers play an important role in nursing students' education and are ultimately their role models, the identification of these behaviors in nursing teachers appears to be essential. This study was conducted to determine nursing students' perceptions of their teachers' uncivil behaviors.

The present study was conducted using a qualitative approach and content analysis. A total of 13 nursing students were selected through purposive sampling, and deep and semi-structured interviews were conducted with them. Content analysis was performed using an inductive approach.

Three main categories were obtained through data analysis; disruptive behaviors affecting communication climate (subthemes: humiliation, the lack of supportiveness, and distrust), disruptive behaviors affecting ethical climate (subthemes: self-centeredness, coercion and aggression, and harassment), and disruptive behaviors affecting learning climate (subthemes: poor teaching skills, poor time management, and indiscipline). Given that human dignity takes precedence over education, any action causing humiliation and embarrassment can have inverse effects on the students and may harm them. These behaviors taint the educational role. Since students select their teachers as their role models, the impact of teachers' uncivil behaviors on students cannot be neglected. Neglecting these behaviors might lead to their persistence in the clinical setting and irreparable damage to patients, who are the ultimate recipients of care.

## Introduction

There has been a dramatic increase in the incidence of uncivil behaviors in nursing education compared to the past (1-3). The majority of students and teachers consider uncivil behaviors in nursing education as a serious threat (4, 5). Destructive uncivil behaviors are serious problems in nursing education that require constant attention and assessment. These behaviors jeopardize the peacefulness of the teachers, students, and the academia (6), have destructive effects on the students and the teachers, and hurt teaching and learning environment and the student-teacher relationship to a degree that might lead to the violation of the rights of all students (3, 7). However, civil behavior is respect for others in the face of disagreement, dispute, and debate (8). These uncivil behaviors cause psychological and physical disturbances for those involved, and might turn into threatening situations if neglected (9). 

Previous studies have reported teachers' uncivil behaviors to manifest themselves in the form of canceling classes without prior notice, coming to class unprepared for teaching, not allowing free discussion in the classroom, acting cold and indifferent, humiliating and scolding the students, rushing through the topics, humiliation, unfairness, imposing their own will, indifference toward the students (3, 10), and being unavailable (4). Amanda has reported that behaviors such as late arrival, coming into the class unprepared, giving dirty looks to impolite students, and disrespecting students provoke retaliation in the students (10). Teachers' uncivil behaviors are not limited to the examples mentioned and also include teaching incompetence (10), immodesty, poor teaching skills (11), and poor communication skills (12, 13). Students have reported experiences of vulnerability (14), helplessness, stress, sleep disorders (15), and depression (16) when exposed to teachers' uncivil behaviors (17-19) and have the notion that teachers wish to get rid of them (8). Teachers may inadvertently provoke retaliation in the students by such uncivil behaviors as late arrival, coming to the class unprepared for learning, giving dirty looks to impolite students, and disrespecting students (20, 21). Some teachers commit uncivil behaviors knowingly or unknowingly, which is proof that it is possible for teachers to cause uncivil behaviors in their colleagues in the academic setting (12). Teachers' manner of responding to the students can exacerbate or abate the tensions (7). Occasionally, they do not realize that the act of humiliating and embarrassing students may unintentionally cause conflict and hostility (22). The conflicts between the teachers and the students disconnect the students from learning process (3), and can deteriorate the learning environment and lead to poor performance and violations in the workplace (23). It is not surprising that people who commit uncivil behaviors in academic settings continue this behavior in the workplace (24). 

Previous studies indicate that teachers are concerned about the growing frequency of these behaviors in nursing education settings (14). These behaviors can disturb those involved, jeopardize the teaching and learning environment, and cause hostility (25). Considering the humanitarian objectives of nursing as a profession, the need for focusing on the ultimate beneficiaries (i.e., the patients), and the importance of developing civility in all communities, especially in nursing education settings, it seems that identifying these behaviors in various societies (including Iran) can provide invaluable information on this topic. To diminish uncivil behaviors, creation of a safe and dependable environment should be given priority (24). The first step is identification of these behaviors, reduction of the conflicts caused by them, understanding of the feelings and perspectives of the students, and hearing their views. Identifying these behaviors and taking measures to reduce them are therefore necessary steps for creating a standard educational setting. The present study was designed to determine students' perceptions of teachers' uncivil behaviors.

## Method

The present study used directed qualitative content analysis and purposive sampling to investigate the perception of nursing students regarding teachers' uncivil behaviors. In this qualitative approach, the phenomena must be investigated in their natural context. The nursing schools of Tehran University of Medical Sciences, Iran University of Medical Sciences, Shahid Beheshti University of Medical Sciences, Islamic Azad University, and Shahed University (Tehran, Iran) were selected. Participants were selected through purposive sampling. The interviews were held in a quiet room in the nursing schools or hospitals. Sampling continued until data saturation, which occurred in the 11^th^ interview, when no further new data could be obtained and previous codes were being repeated. The students had completed at least two semesters of their studies, entered the clinical setting, and received clinical training in nursing schools of the selected medical universities in Tehran. The data extracted from each interview guided the subsequent interviews. After ethical approval and obtaining written informed consent from the participants, in-depth and semi-structured interviews were conducted to collect the data. The researcher first briefed participants on the study objectives, and then, introduced herself at the beginning of each interview and asked participants to fill out and sign informed consent forms for participation in the study and the recording of their voices. Participants were also briefed on the study objectives, the reason for recording the interviews, the voluntary nature of their participation, and the confidentiality of their data and identities. For greater assurance, two more interviews were conducted, which resulted in no new data. Interviews were begun with the questions "Have you witnessed abnormal and disrespectful behaviors from your teachers during your education?", "How did these behaviors affect you?", "On what occasions did you experience these behaviors?" and "Could you describe one of these experiences." In order to obtain more data and clarify certain issues, some probing questions were also asked, and continued with more specific questions. Field notes were taken during the interviews. Each interview lasted between 30 and 60 minutes. All interviews were recorded on tape and the transcripts were typed, reviewed, and coded at the end of each interview. To observe the principle of confidentiality, participants’ names were not revealed. Instead, each of them was given a specific number and their important characteristics such as age, sex, and level of education were recorded, and promptly transcribed verbatim and typed. To immerse in the data, the researcher reviewed the interviews several times and coded them. Data were analyzed simultaneously and continuously by collecting information. Semantic units were extracted in the form of initial codes or open codes from the interviews. The codes were reread several times and placed in subcategories on the basis of similarity and proportion of the participant expressing the same topic. Next, the subcategories were compared with each other and those with similar characteristics were combined to create wider categories, which were presented once more. 

Some of the measures taken to enhance data accuracy included prolonged engrossment in the topic, confirmation of findings by the participants, and observer reviews. To ensure dependability of the data, in addition to the members of the research team, three experts, who were members of the research team, were asked to evaluate the interviews, coding, and categories. To enhance transferability, the entire process of the research and all the work done in the course of the study were prepared in clear and accurate written form to enable others to track and study the population characteristics.

The qualitative content analysis method used in the study included 8 stages proposed by Wildemuth (26). To ensure the confirmability of the data, parts of the interviews were checked along with the extracted codes and subthemes both externally and by 4 postgraduate nursing students familiar with qualitative studies. To allow for future references to the study, the stages and processes of the research were carefully recorded and reported. 

## Results

A total of 13 (10 female and 3 male) students were interviewed. To obtain a wider variety of experiences, maximum variation sampling was used in the selection of participants in terms of their year of study (3^rd^ to 8^th^ semester), age (19-25 years old), and gender.

Participants' rich, in-depth descriptions yielded extraction of 384 initial codes, which were then reduced to 70 after eliminations and integrations at different stages. Finally the 70 codes were converted to 3 themes and 9 subthemes (table 1). Each theme will be explained with its relevant subthemes in the following.

**Table 1:** The themes and subthemes extracted from the interviews

**Theme**	Subtheme
Disruptive behaviors affecting communication climate	Humiliation
	Lack of supportiveness
	Distrust
Disruptive behaviors affecting ethical climate	Self-centeredness
	Coercion and aggression
	Harassment
Disruptive behaviors affecting learning climate	Poor teaching skills
	Poor time management
	Indiscipline

Theme 1:

The first main theme extracted was disruptive behaviors affecting communication climate, which included behaviors that cause the break-up of communication, and the subthemes of humiliation, lack of supportiveness, and distrust.

Humiliation signifies the belittling and disrespecting of others. In the experience of the majority of the students, calling others unpleasant names or attributes taints their self-esteem. Participants emphasized the need for teachers to respect their students. One participant said:

[*During the training, some instructors argued with us in the presence of the patients, and scolded us, and so, the patients would not allow us to care for them, and would tell us that we did not know how to do our job, and humiliated us this way. But the instructor could have simply pulled us aside and told us about our mistake in private*.] (Participant No.1) 

Another participant said: [*One time, one of the students was having difficulty understanding what the teacher was trying to convey, and so, the teacher called him a dumbass, and the student turned beetroot. Is it okay for a teacher to embarrass a student by calling him a dumbass?*] (Participant No. 2)

The next subtheme was lack of supportiveness. The best way for establishing a good relationship with the students is for the teachers to give them emotional support. Participants' experiences showed that teachers' supportiveness enhances the students' self-esteem and motivation for learning; the effect of factors such as pleasantness, openness to criticism, flexibility, fairness, modesty, understanding, and empathy should not be neglected on learning, the communication of knowledge, and the overall development of the student and the university. They considered the teachers' lack of supportiveness and their disregard for the students' wishes as disheartening. One participant asserted: [*The teachers never support us when we need them to, and ignore our problems*.] (Participant No. 5) 

Another subtheme was distrust. Distrust signifies the lack of a strong belief in a person's credibility, honesty, and capability. In the experience of some students, some teachers doubt their students and do not believe their words. One participant said: [*The teachers believe only themselves, and never trust in what their students have to say*.] (Participant No. 4) 

The second main theme extracted was disruptive behavior affecting the ethical climate, which included the subthemes of self-centeredness, coercion and aggression, and harassment.

Self-centeredness is an inimical behavior in which a person considers only his own interest and wishes and never feels accountable toward others. In the experience of some participants, the teachers are unfair and act according to their own impulses. One participant said: [*Some teachers give good grades to the students they like and low grades to those they do not, just to trouble them*.] (Participant No. 1) 

Coercion and aggression was another subtheme of this theme. Coercion signifies forcing others into a situation against their will. In the experience of participants, coercion and aggression lead to poor communication and distanced the students from the teachers out of fear. On the contrary, an amicable and respectful relationship can hearten the students to make greater efforts to resolve their mistakes. One participant said: [*One of the teachers used to literally throw the book at us and shout if we could not give a satisfactory answer to her questions. Of course, we do not know everything; we are here to learn*.] (Participant No. 8)

Harassment was another subtheme, which signifies behaviors that harass others and deprive them of their right to peacefulness. Some participants discussed being harassed by their teachers, which made the girl students particularly uncomfortable. One participant revealed: [*One of our man teachers would give away his phone number, so we would become friends with him*.] (Participant No. 7) Another participant said: [*One of the teachers in our second semester used to get on my nerves with his roving eyes*.] (Participant No. 9) 

The third main theme was disruptive behavior affecting the learning climate, which tends to interfere with appropriate teaching and effective learning, and included the three subthemes of poor teaching skills, poor time management, and indiscipline. One of the subthemes of this theme was poor teaching skills, which signifies the teachers' lack of efforts for promotion of knowledge and skills in others. In the experience of the participants, the teachers' knowledge and teaching techniques are what count in increasing students’ interest in learning. They also asserted that uncreative teaching methods, especially simple lectures, make the class boring and do not motivate the students. One participant argued: [*Some teachers only read the slides out loud, and do not expand on the subject or use modern teaching techniques, which make lectures super boring for us*.] (Participant No. 2) Poor time management was another subtheme of this theme. In the experience of the participants, some teachers do not plan their classes ahead of time and often run out of time by wasting it, and then, request additional sessions. One participant said: [*Teachers spend a great deal of time telling us anecdotes, and so, they run out of time. So, the students get tired and can no longer concentrate. Some teachers keep us longer to finish a topic, and if they cannot, they ask for additional sessions*.] (Participant No. 6) 

The last subtheme of this theme was indiscipline. In the experience of participants, teachers are considered as role models by the students. How can an undisciplined teacher expect discipline from his students? One participant remarked: [*Some teachers arrive late to the classroom*.] (Participant No. 3) 

## Discussion

The results of the present study showed that nursing students experience uncivil behaviors from their teachers in three themes, namely disruptive behaviors affecting communication climate, ethical climate, and learning climate. A study conducted by Clark et al., however, revealed two themes, including destructive behaviors and threatening behaviors (9). A number of the subthemes extracted in the present study show clear differences and similarities with the themes extracted in previous studies, which will be addressed in detail. Humiliation was a subtheme of non-supportive behaviors that was frequently referred to in different forms by the majority of the students interviewed. The students described the major part of being humiliated to have occurred in the form of the teacher ignoring their identity and character or feeling superior to the students and explained that it included becoming the target of the teacher's ridicule, insult, and sarcasm, and not getting responses to their questions. The results of other studies have shown that students consider humiliating behaviors the same as uncivil behaviors and communication as a means of committing civil behaviors (27). Studies conducted in the US by Tantleff-Dunn et al. (7) and Luparell (3) showed that students experienced humiliation as a result of the teacher not responding to their questions and neglecting them. The lack of supportiveness was another subtheme that was experienced in the form of the teacher's general indifference and the lack of attention to the students' requests. Clark and Springer referred to this subtheme as the teacher's refusal to respond to the students' requests (4). Distrust was another subtheme frequently referred to by the students. Although other studies have not directly addressed this subtheme, teachers' inflexibility and bullheadedness were noted several times (28, 29), which may have been due to the teachers' distrust in the students caused by their lying, trickery, and cheating in the exams. In the present study, the teachers' distrust in the students was attributed to the students' dishonesty. 

Self-centeredness was another subtheme discussed by the majority of the students. According to Victor and Cullen, in moral theory, self-centeredness is in line with egoism, which indicates an inconsideration for others (30). In the present study, self-centeredness was manifested as grading the students according to one’s own preferences or presumptions, and in some cases as humiliating the students and making them apologize, which might be due to the teachers' sense of superiority over the students.

Coercion and aggression comprised another subtheme of inimical behaviors, which were said to be manifested in the form of imposing one’s own beliefs, throwing books and arguing with the students. Clark and Carnosso confirm some teachers' tendency to use coercion and aggression on their students (8).

Harassment was another subtheme of inimical behaviors. The students described this behavior as manifesting in the form of the teacher giving his phone number to students of the opposite sex and having roving eyes directed at them. Gallo confirmed some teachers' tendency to harass their students by the cellphone or through emails (11). In the Islamic culture of Iran, interaction between sexes is clearly defined according to the four principles of *Ashram* and *non-Ashram* and *halal* (allowed) and *harm* (forbidden); observing these principles is a duty of every Iranian Muslim. On another note, the main reason for attending university is to acquire knowledge, and interaction with the other sex in the academia is solely for educational purposes, and any friendships that may develop should be toward an educational goal or else be the prelude to achieving educational goals (31). Any behavior or relationship that distances the student from this goal is considered a diversion from the path of communication for the purpose of education (32).

Poor teaching skills of the teacher was a subtheme of threatening behaviors; students referred to the teachers' lack of knowledge, not using different teaching techniques, and reading tedious slides out loud, and also the use of inexperienced teachers. The students believed that teacher's high scientific capabilities and their use of modern teaching techniques creates enthusiasm in them for learning and encourages them to acquire knowledge and skills. Clark also confirms the manifestation of the poor teaching skills of some teachers in the form of using ineffective methods and diversion from the topic of class (33).

Poor time management was another subtheme, which was frequently expressed by the students to take place in the form of wasting time by irrelevant chatter, having no teaching plans, and requests for additional sessions. It is worth noting that no studies were found on this issue, which might be due to the limited number of studies conducted on the topic. The cause may also be that the need for time management is so deeply felt in other countries, that it has become an integral part of their life along with respect for others' rights (34), and the lack of time management has been considered an act of indiscipline (35).

Indiscipline was a subtheme of tainting behaviors, which was manifested as late arrival to class, early dismissal of class, taking care of personal chores inside the class, and not coming to class prepared for teaching. As teachers are role models for their students, their disciplinary conduct makes students observe discipline as a duty and a norm in educational settings. Clark confirmed indiscipline to manifest itself in the form of late arrival to class, early dismissal of the class, and coming to the class unprepared to teach (13).

The results of the present study cannot be taken as fully reflecting the perceptions of the entire community of nursing students in Iran. However, since the university admission system in Iran is centralized rather than localized, and since the education system is also centralized, the same perceptions can be assumed to exist in other parts of the country. The findings of the present study can therefore be indicative of the perceptions of the majority of nursing students across the country.

## Conclusion

The results of the present study indicate that uncivil behaviors in nursing education and their destructive consequences are not issues that can be ignored. Neglecting these abnormal behaviors may lead to aggressive and hostile behaviors. These behaviors might become the norm and form part of the culture of the society over time. Given that teachers play a guiding role for students in addition to their educational role, their contribution to the formation of these behaviors cannot be overlooked. Since the educational environment, and especially teachers, play a major role in fostering committed, ethical, and devoted nurses, and since patients are the ultimate group that benefit from these improvements, it is upon the authorities of the healthcare system to take every necessary measure for promoting civil behaviors and ending the growing trend of uncivil behaviors in nursing education settings for the ultimate purpose of fostering committed and efficient nurses and ensuring patient safety and health. 
